# Why *Um* Helps Auditory Word Recognition: The Temporal Delay Hypothesis

**DOI:** 10.1371/journal.pone.0019792

**Published:** 2011-05-18

**Authors:** Martin Corley, Robert J. Hartsuiker

**Affiliations:** 1 School of Philosophy, Psychology and Language Sciences (PPLS), University of Edinburgh, Edinburgh, United Kingdom; 2 Department of Experimental Psychology, Ghent University, Ghent, Belgium; University of Maribor, Slovenia

## Abstract

Several studies suggest that speech understanding can sometimes benefit from the presence of filled pauses (*uh*, *um*, and the like), and that words following such filled pauses are recognised more quickly. Three experiments examined whether this is because filled pauses serve to delay the onset of upcoming words and these delays facilitate auditory word recognition, or whether the fillers themselves serve to signal upcoming delays in a way which informs listeners' reactions. Participants viewed pairs of images on a computer screen, and followed recorded instructions to press buttons corresponding to either an easy (unmanipulated, with a high-frequency name) or a difficult (visually blurred, low-frequency) image. In all three experiments, participants were faster to respond to easy images. In 50% of trials in each experiment, the name of the image was directly preceded by a delay; in the remaining trials an equivalent delay was included earlier in the instruction. Participants were quicker to respond when a name was directly preceded by a delay, regardless of whether this delay was filled with a spoken *um*, was silent, or contained an artificial tone. This effect did not interact with the effect of image difficulty, nor did it change over the course of each experiment. Taken together, our consistent finding that delays of any kind help word recognition indicates that natural delays such as fillers need not be seen as ‘signals’ to explain the benefits they have to listeners' ability to recognise and respond to the words which follow them.

## Introduction

By far the most common kind of language use is conversation [1]. In conversation, utterances are produced spontaneously. That is, they are “conceived and composed by their speakers even as they are spoken” [2] (p. 136). One consequence of this is that spontaneous speech contains disfluencies. These are generally defined as “phenomena that interrupt the flow of speech and do not add propositional content to an utterance” [3] (p. 709). They include pauses, interruptions (midphrase or midword), repeated words and phrases, restarted sentences, words with elongated pronunciations, such as *the* pronounced *thee* and *a* as *ay*, and fillers such as *uh* and *um*. Such disruptions are very frequent: Averaging across a n*um*ber of studies, and excluding silent hesitations, it has been estimated that disfluency in spontaneous speech affects about 6 per 100 words [3], [4].

At first glance, it would seem that the many disfluencies in spontaneous speech present a formidable challenge to the speech perception system, because disfluencies result in strings of words that are not grammatically correct and strings of sounds that are not words. This view that disfluencies are ‘noise’ and present obstacles to perception [5] (p. 275) is probably the reason that radio broadcasters tend to edit out disfluencies from interviews and that the written media tend to omit disfluencies from their renditions of people's speech [6]. It may also be one of the reasons that most studies on spoken language comprehension have used idealized, fluent utterances. However, the small number of studies that have investigated the effect of disfluencies on word or sentence comprehension converge on the opposite conclusion, namely that under some circumstances, disfluencies can in fact help the listener [5]–[13]. There may be several reasons why disfluencies can facilitate comprehension (see below). This article investigates one property of disfluencies that might make them helpful, namely that they delay the onset of the following word.

Earlier studies have shown benefits of disfluencies on both participants' comprehensibility ratings of sentences and on on-line processing measures. For example, listeners rate utterances including self-repairs as more comprehensible when those repairs are preceded by pauses [13]. In on-line tasks, participants are quicker to identify a ‘correct’ repair word (*orange*) following either a between-word interruption (*yellow*–*orange*), or a mid-word interruption with or without a filler (*yel*–*uh*–*orange*, *yel*–*orange*), compared to fluent controls [5]. The quickest identifications are in cases where the interruption includes a filler. These findings strongly suggest that pauses and fillers help the identification of upcoming words. This conclusion is partially supported by a word-spotting study [6] which shows that both English and Dutch listeners are faster to identify a target word in a carrier sentence when it follows an *uh* in comparison to a control condition without the *uh*. In a comparable set of conditions with or without an *um* there is no effect.

Why would (some) fillers facilitate auditory word recognition? On one account [6], fillers ‘signal’ delays in speech, with specific fillers signaling specific lengths of delay. The fillers could therefore heighten attention for upcoming speech. In particular, this account rests on the assumption that *um* signals a longer delay than *uh* does. This assumption is supported by an analysis of transcriber-rated pause lengths in a speech corpus [14]. Given this difference, *uh* is predicted to signal a relatively short delay, and it is functional for the speech perception system to immediately heighten attention for upcoming speech. But in the case of *um*, heightening attention is less functional, because there is no reason to expect the next word anytime soon[6]. In short, on this account, the speech perception system is sensitive to the patterns of delays that tend to co-occur with fillers in natural speech, so that attention can be allocated in a way that is appropriate to the particular filler that occurs.

However, it is also possible that the benefits for perception emerge from the fact that disfluencies like *uh* and *um*, and any silent pauses preceding or following the filler, considerably delay target word onset themselves. For example, in [6] (Experiment 2) the average duration of *um* was 615 ms; the average durations of preceding and following pauses were 592 ms and 412 ms respectively. According to the *delay hypothesis* tested here, temporal delay facilitates word recognition. Anecdotal evidence suggests that speakers who are difficult to follow will be easier to understand when they speak more slowly (presumably, slowing speech affects pausing). Furthermore, there are theoretical reasons why a delay in word onset might facilitate word recognition. For example, delays might make the listener's segmentation problem easier, because speech sounds spanning a delay most likely do not belong to the same word. It is also possible that delays help top-down processes: The more time passes, the more time there is to make top-down predictions about the next word. Finally, it may be the case that attention builds up over the course of any delay.

The delay hypothesis predicts that *um*, like *uh*, should aid word recognition and therefore appears to conflict with previous findings [6]. But as noted above, in that study there were relatively lengthy silent pauses preceding and following the *uhs* and *um*s, and these pauses were left intact in the control stimuli. As a result, there were delays in the disfluent stimuli, but also in the ‘fluent’ stimuli. According to the delay hypothesis, these silent pauses in the fluent stimuli would themselves facilitate word recognition, so that the further delay from having a filler would do little to facilitate this process even more.

To test the delay hypothesis, we conducted three experiments that assessed the effects of three types of delays on auditory word recognition. In all experiments, listeners viewed displays of two images and listened to instructions to press a button corresponding to one of them. In the delay conditions, listeners heard an instruction with a delay immediately before the target word. In the control conditions, the instruction also contained a delay, but earlier in the sentence. This led to instructions like those in (1).

(1a) Now press the button for the <delay> <target>, please

(1b) Now press the <delay> button for the <target>, please

We opted for a control condition with an early delay, so that the total time to target was constant across conditions, and so that both experimental and control stimuli contained exactly the same auditory materials (albeit in a different order). Thus, any difference between experimental and control conditions can only be ascribed to the difference in the position of the delay. Note that instructions with an invariant syntactic structure and invariant lexical content (bar the target item) have also been used in eyetracking experiments [15]. Additionally, to ensure maximal comparability across experiments we used the same acoustic token of the carrier sentence, and delays of exactly the same length in every condition of all experiments.

Experiment 1 examined the effect of a naturalistic disfluency, namely *um*. Experiment 2 tested whether any facilitative effect on word recognition is a specific property of fillers like *um*, or whether silent pauses can also help word recognition (as predicted by the delay hypothesis). Experiment 3 tested whether there is also facilitation when the delay is clearly not a naturalistic disfluency. We therefore used an artificially generated sine wave tone.

In each experiment, upon hearing an auditory instruction naming one of two depicted objects, participants had to respond by pressing one of two buttons (corresponding to the left-hand or right-hand object). Half of the instructions included a late delay, just before the object was named, and the other half included an earlier delay. To establish that the paradigm had sufficient power to reveal reaction time effects on auditory word recognition, each experiment also included a task difficulty manipulation. In the difficult condition, the target picture had a low-frequency (LF) name and was visually blurred. In the easy condition, the target picture had a high-frequency (HF) name and was visually intact. Thus the two factors manipulated orthogonally in a within-participants design were delay and task difficulty. In each experiment, recordings were made of times (relative to the onset of the target item name) taken for accurate responses to the instructions.

## Results

We analysed our experiments using Generalized Linear Mixed-Effects models. Effects, and their probabilities, were estimated using 10,000 Markov chain Monte Carlo samples.

### Experiment 1: Early vs. late *um*

Table 1 shows the participant mean correct reaction times. There was a main effect of delay, with participants 32 ms faster to respond in the delay (late *um*) condition (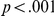). There was a separate effect of task difficulty, with participants taking 63 ms longer to respond to blurred LF items (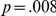). Reaction time also decreased over the experiment, by an estimated mean of 1.5 ms per trial (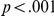). In this experiment there was a marginal interaction of trial number with frequency (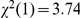, 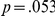), such that responses to blurred images with LF names speeded up by an additional 0.8 ms per trial (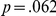). There were no other significant interactions (all 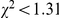, 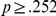).

**Table 1:** Experiment 1.

Instructions	Target Type	
	clear HF	blurred LF
control (*um* early)	703 (29.1)	746 (29.0)
delay (*um* late)	674 (27.0)	712 (26.6)

Participant mean correct RT (ms), relative to target onset (SE in brackets).

Experiment 1 showed a clear recognition advantage when the word followed a local delay which was filled with an *um* (a pilot study showed a similar delay advantage in a comparison of the same late *um* condition as reported here with a completely fluent condition). The results extend those of Fox Tree [6] by showing that an *um* before a target can help recognition of that target, just as *uh* can. The findings are consistent with the delay hypothesis, but it is possible of course that the facilitation resulted from the nature of the delay (i.e., a delay containing a filler) rather than from the delay itself. Experiment 2 therefore substituted *um* with silence.

### Experiment 2: Early vs. late silences

In this experiment, each *um* in the materials of Experiment 1 was replaced with a silence of the same length, resulting in auditory stimuli with a silence before the word *button* in the control condition, or before the target in the delay condition.

Table 2 shows the mean correct reaction times by participants. There was a main effect of delay, with participants 27 ms faster to respond in the delay (late *um*) condition (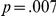). The effect of task difficulty was also significant, with blurred LF items resulting in responses which were 51 ms longer (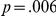). Reaction time decreased over the experiment by an estimated mean of 2.9 ms per trial (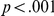). None of these effects interacted with each other (all 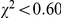, 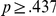).

**Table 2:** Experiment 2.

Instructions	Target Type	
	clear HF	blurred LF
Control (silence early)	612 (40.5)	652 (44.7)
delay (silence late)	568 (43.2)	631 (46.6)

Participant mean correct RT (ms), relative to target onset (SE in brackets).

Experiment 2 showed that a silent pause immediately before a target word affects listeners in the same way as *um* did in the previous experiment, providing further evidence for the delay hypothesis. One might argue, however, that the facilitatory effect did not result from the delay, but from the fact that silent pauses, just like *uhs* and *ums*, occur as naturalistic disfluencies. Silent pauses fulfill several functions in discourse [16] and it is conceivable that they, just like *uh* and *um*, sometimes co-occur with production difficulties and therefore increase listeners' attention to the following word. We therefore put the delay hypothesis to the test again, but now used sounds that cannot be reasonably interpreted as naturalistic disfluencies, namely artificial tones (consisting of sine waves). The delay hypothesis predicts that tones directly preceding the target will still facilitate recognition, but any account on which the facilitation in the previous experiments results from listeners interpreting the content of delays as a signal predicts no effect.

### Experiment 3: Early vs. late tones

Experiment 3 was a replication of Experiments 1–2 using delays filled with non-speech sounds. Each *um* in the original materials used for Experiment 1 was replaced with a sine wave tone of the same duration, and with a frequency (400 Hz) chosen to approximate the baseline frequency of the recorded speaker.

Table 3 shows the mean correct reaction times by participants. There was a main effect of delay, with participants 56 ms faster to respond in the delay (late *um*) condition (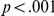). The effect of task difficulty was also significant, with blurred LF items resulting in responses which were 44 ms longer (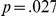). Reaction time decreased over the experiment by an estimated mean of 1.1 ms per trial (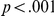). None of these effects interacted with each other (all 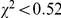, 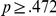).

**Table 3:** Experiment 3.

Instructions	Target Type	
	clear HF	blurred LF
Control (tone early)	638 (43.1)	676 (45.1)
delay (tone late)	575 (37.5)	630 (40.8)

Participant mean correct RT (ms), relative to target onset (SE in brackets).

### Cross-experiment comparison

All three experiments showed a delay advantage, and the numerical magnitude of this advantage was comparable for each kind of delay: *um* (Experiment 1): 32 ms, silence (Experiment 2): 27 ms, tone (Experiment 3): 56 ms. To test whether there was a differential delay advantage for the different types of delay, we conducted a further analysis, incorporating an additional ‘experiment’ factor (corresponding to type of delay). There was an interaction of trial number with experiment (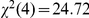, 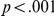), corresponding to differences in the per-trial speedup reported above. Critically, however, there was no interaction between delay and experiment (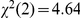, 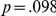), showing that there were no discernable differences in participants' responses to the different types of delay.

## Discussion

In each of three experiments we found clear effects of delays on word recognition. It did not matter whether such delays were filled with *um* (Experiment 1), were silent (Experiment 2), or were filled with a tone that was clearly not part of speech (Experiment 3). A conclusion that stands out from this work is that any delay in word onset can help word recognition. This has an important theoretical implication. The current data certainly do not rule out that listeners are sensitive to the distributional properties of speech following fillers like *uh*, *um*, and the like, so that they can predict when the next word will follow or even what word will follow. However, our data do show that it is not necessary to postulate such sensitivity. The benefits of fillers on word recognition can just as easily be explained in terms of the delay that such fillers create.

Our findings contrast with an earlier study by Fox Tree which showed an *uh*-advantage, but no *um*-advantage in a word-spotting task[6]. This was interpreted as a consequence of the differences in delay that *uh* and *um* would signal[6], [14]. However, the present study did find an effect of *um*. A possible reason for this difference in findings is that the *ums* in Fox Tree's study were preceded and followed by lengthy silent pauses, which were left in the “fluent” control stimuli. Our Experiment 2 shows that silent delays also facilitate word recognition. This may have masked any effect of *um* in the earlier study.

It is true of course that our study differed from that of Fox Tree in several further ways. The most striking difference is probably that Fox Tree used naturalistic utterances, with different carrier sentences and different *uhs* and *ums* on every disfluent trial, whereas we used one and the same carrier sentence, and always used the same delay (i.e., same *um*, silence, or tone). Fox Tree's choice of stimuli undoubtedly promoted ecological validity more than ours, but this of course traded-off with experimental control. Whereas our design allows us to directly compare the effects of different types of delay (because everything else was held constant across experiments, except for the random factor participants), it is rather difficult to directly compare Fox Tree's *uh* and *um* conditions, because the carrier sentences and target words were different, the *uhs* and *ums* varied in length, and the pauses before and after *um* varied too[6].

Two sets of studies have, however, used different types of delays in circumstances which allow for direct comparison of their effects. In one study, participants were asked to judge the grammaticality of recorded utterances which included spoken disfluencies (*uh uh*) or “environmental noises” (such as dog barks) in positions which were expected either to facilitate or interfere with understanding [17]. In each of two experiments, the effects of environmental noise were shown to be effectively the same as those of disfluency. A second set of studies examined ERP responses to words following silent [18] and disfluent *uh* [10] delays. In each case, the N400 component associated with contextually less predictable words was attenuated following a delay. Taken together, these studies suggest that different types of hesitation can be shown to have similar effects across a variety of paradigms and materials, providing converging evidence that the form of a delay to the spoken signal may be less important than the time taken.

One potential caveat with such studies is that they tend to repeat acoustic tokens in order to achieve experimental control. In the present study our use of a fixed token of the carrier sentence, and a fixed token of *um*, may have led the participants to process the *um* and following words in ways that differ from the normal listening situation. Specifically, in normal listening situations, listeners might interpret *um* and the like as a signal of upcoming delay (which heightens attention in ways appropriate for the ‘meanings’ of the particular disfluencies). However, because in our experiments the target word almost immediately followed the *um*, and because listener sensitivity to session-local distributional properties of *um* would gradually overwrite their global sensitivities, listeners would, in the course of a session, stop expecting a further delay following *um*. Including the effects of trial number in our analyses provides a direct test of whether this caveat threatens our conclusions. Specifically, any account on which listeners pick up on the properties of our materials would need to further assume that the effects of delays change over the course of the experimental session. As is often the case in reaction time experiments, the analyses showed that responses became somewhat faster over the course of the experiment, but in none of the experiments was there an interaction between item number and delay, showing that participants' responses to delays did not change over time.

Our main conclusion is that delays in word onset facilitate word recognition, and that such facilitation is independent of the type of delay. There are several reasons why a delay in itself might help. On one account, delays help low-level speech segmentation processes. Because running speech often contains no clear word boundaries, the segmentation process has to figure out where one word ends and the next one begins, as illustrated by the classic example *I scream for ice cream*. Obviously, this segmentation problem can be reduced when there are delays between words. However, we do not think a segmentation account can explain our results. In particular, in our experimental condition, the *um* always occurred between *the* and the target item. If the segmentation account is correct, then listeners should encounter difficulties segmenting the string of sounds consisting of *the* and the initial sound(s) of the target item. The cohort of words starting with *the*, however, is very small, and only contains words in which the schwa is followed by a /t/ (e.g., *that*), an /m/ (*themselves*), or a /w/ (*the one*). Only one target word (*tree*) started with one of those consonants and could therefore have led to segmentation problems.

Another reason why delay helps word recognition may be that delay allows time for any top-down processes to affect recognition processes. Visual-world eyetracking experiments suggest that listeners, when hearing speech in a visual context, make linguistic predictions about upcoming references [19]–[21]. Given the ubiquity of NPs consisting of a determiner and noun in the language, it is possible that determiners lead to the prediction of nouns (and help subsequent identification of nouns), and that these predictions become more effective as more time passes.

Finally, it is possible that delays do not affect the mechanisms of word recognition themselves, but affect an attentional modulation of recognition processes. On such an account, any delay in speech will lead to a transient increase in attention, so that the next word can be more readily identified. Consistent with this account, it has been shown that stimuli containing an *uh*, as compared to fluent controls, modulated the amplitude of the mismatch negativity and P300 components in the ERP signal[9]. It is well established that these components are sensitive to variations in attention. Additionally, in subsequent memory tests, words that had followed *uh* were recalled better than control words, which is again consistent with an attentional account[9], [10].

Our findings support the perhaps counterintuitive conclusion that fillers like *um* can sometimes help (rather than hinder) listeners to identify spoken words. But critically, the data show that the same is true for silent pauses and pauses filled with artificially generated tones. It thus seems unnecessary to postulate listener sensitivity to the distributional properties of pause durations after fillers to explain why fillers help.

## Materials and Methods

### Ethics Statement

Ethical approval for this research was obtained from the Ethics Committee of PPLS, University of Edinburgh, in accordance with the guidelines of the British Psychological Society. As approved by the committee, participants were informed of their right to withdraw and gave verbal consent to take part in the study. Since the data were analysed anonymously, signed informed consent was not obtained.

### Materials

The experimental materials consisted of auditory and visual stimuli. The latter were pairs of pictures with high- and low-frequency names. The pictures were a subset of Rossion and Pourtois' colored versions of a standardized picture set [22], [23], and were normed for (lemma) frequency, visual complexity, and familiarity. Two groups of 16 pictures were generated: 16 HF pictures (mean name frequency 300 occurrences per million in the CELEX database [24]; range 153–796) and 16 LF pictures (mean name frequency 5.29; range 0.22–9.89). Each of the LF pictures was blurred with an image processor using a radius setting of 15 pixels. The resulting LF blurred pictures were paired four times with the HF pictures (never in the same combinations), resulting in 64 picture pairs (see (2) for a list of pictures used). Three pairs of mid-frequency items (lamp-cake, clock-knife, wheel-cow) were used for practice trials at the start of the experiment. No picture depicted a word that started with a vowel (because this would be preceded by thee in an instruction, which could be confused with a disfluency), and no pair of pictures represented words that began with the same phoneme, or had semantic overlap. Each individual picture was on the left of the screen for two of the four times it appeared, and on the right for the remainder. It was a target twice: once each for a late *um* and an early *um* instruction, once on each side of the screen.

(2a) *Pictures in the difficult (LF) condition:* broom; clown; flute; frog; harp; kite; peach; pear; rake; saw; skunk; sledge; snail; spool; swan; vase.

(2b) *Pictures in the easy (HF) condition:* bed; book; car; church; door; dress; fish; foot; hair; hand; heart; house; leg; mouth; sun; tree.

The auditory stimuli consisted of instructions to press a button corresponding to a particular picture. They were always of the form *now press the button for the um <target>*, *please* (delay [late *um*] condition) and *now press the um button for the <target>*, *please* (control [early *um*] condition). There were two versions of each instruction for each picture in the set of 32: one containing a late *um* and one containing an early *um* one. This resulted in 64 utterances in total.

To make the recordings, a female native speaker of English read a list with each target item embedded in the template instruction sentence (see above). After the recording, all target items were removed from their original contexts, together with the word *please* which followed them, and spliced into one version of the carrier sentence which had not originally included any of the target items. This resulted in a set of 32 fluent instructions, for each of which the target word onset was exactly 1219 ms after the utterance onset. To create the delay (late *um*) and control (early *um*) instructions, the speaker was asked to read a number of instructions referring to low-frequency items, inserting an *um* “as naturally as possible”.

For Experiment 1, the single *um* that we judged the most natural was selected, and was spliced into two copies of the fluent instructions, immediately before the target word (delay condition) for the first copy, and immediately before the word *button* (control condition) for the second copy. The *um* in each sentence was 1078 ms long. For Experiment 2, the *ums* were replaced with silences of the same length, resulting in auditory stimuli with a 1078 ms silence before the word *button* in the control condition, or before the target in the delay condition. For Experiment 3, each *um* in the materials used for Experiment 1 was replaced with a sine wave tone of exactly the same duration. The frequency of the tone (400 Hz) was chosen to approximate the baseline frequency of the recorded speaker. Finally, each recording was converted to a 16-bit 22050 Hz WAV file, for use with E-Prime experimental software.

### Methods

Participants were tested individually in a quiet room. They were informed that they were participating in an experiment on sentence comprehension, and that they would be listening to a series of recordings of a speaker giving instructions as fast as possible. The aim of the study was purportedly to establish how easy it is to follow instructions given in stressful situations. This minor deception was necessary to justify the disfluencies in the study. Participants were instructed that they would be presented with a series of displays of picture pairs. Each pair would be accompanied by instructions to press the button corresponding to a given object. Participants had a 5-button response-box in front of them: If the picture referred to was on the right, they were to press the rightmost button; if on the left, the leftmost button. It was stressed that they should respond as quickly as possible, without losing accuracy.

Prior to the experiment, three practice items allowed the participants to familiarize themselves with the procedure, and to adjust the volume on the headphones they wore to hear the instructions. The practice session was identical to the experimental session in all respects bar one: The 3 practice items were always presented in a fixed sequence, whereas the presentation order of the 64 experimental items was randomized.

In the practice session as well as in the experiment proper, each display of a picture pair was preceded by a ‘+’, which remained on the screen for 200 ms, to signal that a new pair of pictures was about to come up. The pictures followed this display immediately. At the same time as the pictures appeared, the corresponding instruction was played. Each instruction was played in full, regardless of whether or not a button had been pressed before it ended. The instructions always finished before the pictures were removed, 4 seconds after onset. Once each trial had finished, the screen was blanked, and the next trial began with a ‘+’ after a 250 ms pause. The time between the onset of the instruction and the corresponding button press was recorded for each correct response. Prior to analysis, all correct reaction times were converted to times relative to the target onset. Since each *um*, silence, or tone was 1078 ms long, in all experiments the target onset in both conditions occurred 2297 ms after the utterance onset, and 2297 ms was accordingly subtracted from all recorded latencies.

Participants were all students at the University of Edinburgh. In Experiment 1, 35 participants made errors on 36 trials (1.9% of the data). In Experiment 2, 16 participants made errors on 18 trials (1.8%); In Experiment 3, 15 participants made errors on 22 trials (2.2%). All errorful responses were excluded from further analysis.

Analyses were carried out by fitting Generalized Linear Mixed-Effects models, as implemented in the lme4 library in R [25], [26]. Such analyses handle each trial as a separate data point, allowing for the inclusion of trial number as a fixed effect. In each of our analyses, we started with a base model including random per-participant and per-item variation. We then added predictors of interest, evaluating each predictor's contribution to the model using 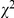 likelihood-ratio tests, until no further predictor or interaction improved the model fit. Coefficients in the saturated model were estimated using 10,000 Markov chain Monte Carlo samples [25], [27].
